# Six Tissue Transcriptomics Reveals Specific Immune Suppression in Spleen by Dietary Polyunsaturated Fatty Acids

**DOI:** 10.1371/journal.pone.0155099

**Published:** 2016-05-11

**Authors:** Sara L. Svahn, Leif Väremo, Britt G. Gabrielsson, Eduard Peris, Intawat Nookaew, Louise Grahnemo, Ann-Sofie Sandberg, Ingrid Wernstedt Asterholm, John-Olov Jansson, Jens Nielsen, Maria E. Johansson

**Affiliations:** 1 Dept. of Physiology, Institute of Neuroscience and Physiology, Gothenburg, Sweden; 2 Dept. of Biology and Biological Engineering, Chalmers University of Technology, Gothenburg, Sweden; 3 Comparative Genomics Group, Biosciences Division, Oak Ridge National Laboratory, Oak Ridge, Tennessee, United States of America; 4 Dept. of Rheumatology and Inflammation Research, Gothenburg, Sweden; Hosptial Infantil Universitario Niño Jesús, CIBEROBN, SPAIN

## Abstract

Dietary polyunsaturated fatty acids (PUFA) are suggested to modulate immune function, but the effects of dietary fatty acids composition on gene expression patterns in immune organs have not been fully characterized. In the current study we investigated how dietary fatty acids composition affects the total transcriptome profile, and especially, immune related genes in two immune organs, spleen (SPL) and bone marrow cells (BMC). Four tissues with metabolic function, skeletal muscle (SKM), white adipose tissue (WAT), brown adipose tissue (BAT), and liver (LIV), were investigated as a comparison. Following 8 weeks on low fat diet (LFD), high fat diet (HFD) rich in saturated fatty acids (HFD-S), or HFD rich in PUFA (HFD-P), tissue transcriptomics were analyzed by microarray and metabolic health assessed by fasting blood glucose level, HOMA-IR index, oral glucose tolerance test as well as quantification of crown-like structures in WAT. HFD-P corrected the metabolic phenotype induced by HFD-S. Interestingly, SKM and BMC were relatively inert to the diets, whereas the two adipose tissues (WAT and BAT) were mainly affected by HFD *per se* (both HFD-S and HFD-P). In particular, WAT gene expression was driven closer to that of the immune organs SPL and BMC by HFDs. The LIV exhibited different responses to both of the HFDs. Surprisingly, the spleen showed a major response to HFD-P (82 genes differed from LFD, mostly immune genes), while it was not affected at all by HFD-S (0 genes differed from LFD). In conclusion, the quantity and composition of dietary fatty acids affected the transcriptome in distinct manners in different organs. Remarkably, dietary PUFA, but not saturated fat, prompted a specific regulation of immune related genes in the spleen, opening the possibility that PUFA can regulate immune function by influencing gene expression in this organ.

## Introduction

Fatty acids are a heterogeneous group of macronutrients that can be divided into saturated fatty acids (SFA), monounsaturated fatty acids (MUFA) and polyunsaturated fatty acids (PUFA). It has been shown that these three types of fat affect the body and the immune system differently, especially in the pathological inflammatory responses associated with metabolic disease [1,2,3].

Previously, low fat diets (LFDs) were widely recommended to maintain a healthy body weight. Current dietary recommendations are however less focused on the amount of fat and more on the fatty acid composition [4,5]. Interestingly, in several observational studies, the amount of dietary fat has only been associated with minor or negligible effects on cardiometabolic diseases, such as cardiovascular diseases and type-2 diabetes mellitus, while the dietary fatty acid composition appears to be more important [5,6].

SFA are typically categorized as unhealthy, partly because they contribute to low-grade chronic inflammation [1]. For example, it has been shown that replacing the energy from SFA with the equivalent energy from unsaturated fatty acids reduces the risk of developing coronary heart disease [5,6].

PUFA are mainly divided into omega-3 (ω-3 PUFA) and omega-6 fatty acids (ω-6 PUFA). ω-3 PUFA are regarded as anti-inflammatory, whereas ω-6 PUFA, especially arachidonic acid, are essentially regarded as pro-inflammatory [3]. Recently, we showed that the dietary fatty acid composition greatly affected the survival of mice subjected to *S*. *aureus*-induced sepsis [7,8]. Mice fed high fat diet (HFD) rich in SFA (HFD-S) had decreased survival and increased bacterial load compared with mice fed HFD rich in PUFA (HFD-P). Neutrophils are of major importance for early defense against bacterial infections. Therefore, the difference in survival between mice fed HFD-S and HFD-P was attributed to an increase in neutrophil frequency in the bone marrow from mice fed HFD-P prior to the infection.

To better understand the potential benefits of dietary fatty acid composition, it is important to understand the underlying mechanisms of the different diets. Several studies have investigated the dietary effect on the transcriptome profile of single tissues [9,10]. However, the number of studies investigating and comparing the transcriptome profile in several metabolic and immune-related organs, using the same analysis strategy for all tissues, is limited. In the present study, we investigated how the fatty acid composition affected the total transcriptome profile, and especially the immune related genes, in the immune organs spleen (SPL) and bone marrow cells (BMC). Since PUFA has major effects on metabolic tissues [11,12] we also included metabolic organs; skeletal muscle (SKM), white adipose tissue (WAT), brown adipose tissue (BAT), and liver (LIV), to be able to compare the gene expression pattern in both immune and metabolic tissues.

## Materials and Methods

### Experiment protocols, housing and diets

Six-week old, male, C57BL/6 mice were obtained from Harlan Netherlands B.V. (Horst, The Netherlands). The mice were housed under standard conditions of light and temperature at the animal facility of the Laboratory for Experimental Biomedicine at University of Gothenburg, Sweden. Water and food were provided *ad libitum*. At the age of 7 weeks, the mice were randomized into one of the following diets: LFD (D12450B, 3.9 kcal/g, 10 kcal% fat, 20 kcal% protein, 70% carbohydrate, Research Diets, New Brunswick, NJ, USA); HFD-S (D12492, 5.2 kcal/g, 60 kcal% fat, 20 kcal% protein, 20 kcal% carbohydrate, Research Diets) and HFD-P (D09020505; same composition as HFD-S of fat, protein, and carbohydrate, but 69% of the lard was exchanged for menhaden oil; Research Diets). The (ω-6 PUFAs)/(ω-3 PUFAs) ratio was 14.2 (29.9/2.1) in HFD-S, 0.6 (16.2/25.6) in HFD-P and 8.2 (42.4/5.2) in LFD (LFD had, however, considerably less total fat compared with the two HFDs). The diets were matched to have similar macronutrient sources except for the fat. The composition of the diets is shown in Table 1.

**Table 1:** Energy density and composition of experimental diets.

	LFD	HFD-S	HFD-P
Energy density (kcal/g)	3.9	5.2	5.2
Macronutrients (% kcal)			
Protein	20	20	20
Carbohydrate	70	20	20
Fat	10	60	60
Fat source (% of total fat)			
Soybean oil	55.6	9.3	9.3
Lard	44.4	90.7	27.8
Menhaden oil	-	-	63.0
Fatty acids (% by wt of total fatty acids)			
∑ SFA	22.7	32.0	28.7
∑ MUFA	29.8	36.0	27.5
∑ PUFA	47.5	32.0	43.9
∑ ω-3 total fat	5.2	2.1	25.6
∑ ω-6 total fat	42.4	29.9	16.2
ω-6/ω-3	8.2	14.2	0.6

SFA, saturated fatty acids, MUFA; monounsaturated fatty acids; PUFA, polyunsaturated fatty acids; ω-3, omega-3 fatty acids; ω-6, omega-6 fatty acids; LFD, low fat diet; HFD-S, high fat diet rich in saturated fatty acids; HFD-P, high fat diet rich in polyunsaturated fatty acids.

After 8 weeks of diet intervention, the animals were anesthetized and killed by cardiac puncture. Tissues were rapidly dissected out, weighed and snap frozen in liquid nitrogen. The samples were kept at -80°C until further analyses. All experiments were approved in advance by the ethics committee for animal care of the University of Gothenburg.

### RNA isolation and microarray analysis

Total RNA from SKM (gastrocnemius), BMC, WAT (retroperitoneal), BAT (intrascapular), SPL and LIV were isolated using the RNeasy Lipid Tissue Mini kit (BMC, WAT, BAT, SPL LIV; Qiagen Nordic, Sweden) or the RNeasy Fibrous Tissue Mini (SKM; Qiagen) according to the manufacturer’s instruction. Total RNA concentration was measured by NanoDrop (Thermo Fisher Scientific, Gothenburg, Sweden). The quality of the RNA was evaluated by RNA 6000 Nano LabChip for Agilent 2100 Bioanalyzer (Agilent Technologies, Sweden). The RNA was labeled and hybridized to GeneChip^®^ Mouse Gene 1 x ST arrays (Affymetrix, Santa Clara, CA, USA) at the genomics core facility, Swegene Centre for Integrative Biology at Lund University, Sweden.

### Real-time Quantitative Polymerase Chain Reaction Assays in WAT

To evaluate mRNA expression, retroperitoneal WAT was homogenized using Isol-RNA lysis reagent and chloroform (5PRIME, Hilden, Germany) with Tissuelyser II (Qiagen Nordic, Sollentuna, Sweden) and 5 mm SS beads. The aqueous phase was further purified using ReliaPrep RNA Miniprep System (Promega, Madison, WI, USA). RNA was converted into cDNA using the qScript kit (Quantan BioScience, Gaithersburg, MD, USA).

Gene expression levels of macrophage markers *Adgre1* (F4/80), *Cd40* (CD40) and *Ccl2* (MCP-1) were measured by qRT-PCR, which was carried out using fast SYBR Green Master Mix (Applied Biosystems, Waltham, MA, USA) and analyzed on the QuantStudio 7 Flex Real-Time PCR instrument (Applied Biosystems). Primer sequences for each gene were as follows: *Adgre1* forward 5'- CTTTGGCTATGGGCTTCCAGTC-3', reverse 5'- GCAAGGAGGACAGAGTTTATCGTG-3', *Cd40* forward 5'-TTGTTGACAGCGGTCCATCTA-3', reverse 5'-CCATCGTGGAGGTACTGTTTG-3', *Ccl2* forward 5’-ACTGAAGCCAGCTCTCTCTTCC-3’, reverse 5’-TTCCTTCTTGGGGTCAGCACAG-3’ and *Actb* forward 5'-GACCCAGATCATGTTTGAGA-3', reverse 5'-GAGCATAGCCCTCGTAGAT-3.

A melting curve analysis was performed in each experiment for all genes to confirm specificity of single-target amplification. All samples were amplified in duplicate and analyses were performed under nuclease-free conditions. Gene-expression levels were calculated using the comparative CT with *Actb* as endogenous control [13].

### Histology staining and Immunohistochemistry

#### ORO staining of LIV

LIV used for Oil red O (ORO) staining were rapidly dissected and stored in 6% buffered formaldehyde (Histolab, Gothenburg, Sweden) for 48 hours prior to sucrose treatment for dehydration. Afterwards, the samples were embedded in OCT and subsequently sectioned followed by staining with ORO and hematoxylin to assess lipid content and morphology. ORO area was computed by an image analysis program (CellSens Dimension Desktop 1.5, Version 1.5, Olympus Optical Company, Hamburg, Germany). Representative micrographs were captured with an Olympus BX60F5 microscope with a 10X objective, connected to an Olympus DP72 camera.

#### F4/80 staining of WAT

Macrophages infiltrating WAT arrange around dead adipocytes, forming crown-like structures (CLS) [14], therefore we stained WAT for macrophage marker F4/80. Adipose tissue was fixed in 4% formaldehyde, embedded in paraffin and sectioned. Sections were then deparaffinized and rehydrated. Antigen retrieval was performed by incubation in citrate buffer, pH 6.0, for 15 minutes, and endogenous biotin was blocked using avidin/biotin blocking kit (Vector Laboratories, Burlingame, CA, USA) for 15 minutes. Endogenous peroxidase activity was quenched by 30 minutes incubation in 0.6% hydrogen peroxide. Staining was performed using a primary F4/80 rat anti-mouse antibody (1:20, AbD Serotec, Raleigh, NC, USA) followed by a biotinylated rabbit anti-rat secondary antibody (1:200, Vector Laboratories). Binding of secondary antibody was visualized using an avidin biotinylated-horseradish peroxidase complex (Vector Laboratories) followed by DAB staining (Dako, Glostrup, Denmark). Sections were counterstained with Mayers hematoxylin. Images were obtained with a MIRAX Scan (Carl Zeiss, Göttingen, Germany) and analyses were done using BioPix iQ software (version 2.1.4., BioPix, Göteborg, Sweden). Representative micrographs were captured with an Olympus BX60F5 microscope with a 10X objective, connected to an Olympus DP72 camera.

### Oral glucose tolerance test and the homeostasis model assessment

The glucose clearance after an oral bolus dose of D-glucose (Sigma-Aldrich, St. Louis, MO, USA) was estimated by an oral glucose tolerance test (OGTT). Plasma glucose levels were measured in blood from the tail vein (Accu-Chek, Roche, Mannheim, Germany) in mice fasted for 3 hours and then at 15, 30, 45, 60 and 120 minutes after an oral dose of glucose (3 g/kg; 6 μl/g body weight). The blood glucose and serum insulin levels were measured, and the homeostasis model assessment of insulin resistance (HOMA-IR) index was calculated using the formula: fasting blood glucose [mmol/L]×fasting serum insulin [μU/mL]/22.5 [15].

### Statistical analysis

#### Analysis of microarray data

The initial dataset consisted of 74 microarrays from the six tissues (SKM, BMC, WAT, BAT, SPL and LIV). After quality control, one microarray from SPL was removed from the analysis, as it appeared to be an outlier in a principle component analysis (PCA) (S1 Fig). The final transcriptional analysis was performed on the remaining 73 microarrays: 4 biological replicates per tissue and diet except for LIV/LFD and LIV/HFD-S with 5 replicates each and SPL/LFD with 3 replicates. The data are available at the Gene Expression Omnibus (GEO), accession GSE79434.

All analysis related to the microarray data was carried out in the statistical software environment R. The raw microarray data was preprocessed using iterPLIER and quantile normalization and reduced to contain only probe sets belonging to the main design category. A differential expression analysis for the three possible pair-wise comparisons between diets, within each tissue, was carried out using the Bioconductor R package piano [16,17] which uses the functionality provided by the Bioconductor R package limma [18]. The *p*-values for the genes were adjusted for multiple testing using the method of Benjamini and Hochberg and are referred to as *q*-values.

#### Gene-set analysis of immune system Gene Ontology-terms

Because our previous findings demonstrate that fatty acids affect the immune system, in this paper, we have focused on the Gene Ontology (GO)-terms under the parent GO-term “immune system process”. We define this group of GO-terms as “immune system GO-terms”. Piano was used for performing gene-set analysis of these immune system GO-terms, excluding gene-sets with less than 10 or more than 500 genes. The p-values for the gene-sets were adjusted for multiple testing using the method of Benjamini and Hochberg and are referred to as q-values. The heatmap in Fig 3 is based on significant GO-terms (q<0.05 in at least one comparison) according to the so called non-directional class in piano. Further on, the GO-terms were color-coded to reflect the underlying transcriptional changes, based on the so called distinct- and mixed-directional classes in piano.

#### Random Forest classification

The random forest classification was carried out in R using the random Forest package. Each sample was classified as belonging to either cluster 1 or 2 based on a model trained on the remaining samples (leave-one-out cross-validation). During the training of each model (one per sample), the importance of individual probe sets for the classification were extracted. The importance was given by a score called the mean decrease in Gini index (Gini coefficient). The top 200 important probe sets (defined by having the highest average Gini coefficients) were used in a GO-term (all Biological Processes) overrepresentation analysis (hypergeometric test using piano). To find the top 12 most important genes for classification, the classification procedure was repeated 100 times, i.e. the class of each sample was predicted 100 times based on 100 different models/sample. The average Gini coefficient over all repeated runs could then be used to rank the genes by importance more robustly than from 1 run.

#### Gene-set analysis of NFκB transcription factor gene-sets

Transcription factor gene-sets from TRANSFAC [19] and JASPAR [20] were downloaded from the Enrichr [21] website (TRANSFAC_and_JASPAR_PWMs.txt) and from the Molecular Signature Database [22] (c3.tft.v5.0.symbols.gmt). Gene-sets representing nuclear factor kappa-light-chain-enhancer of activated B cells (NFκB) where then extracted from these gene-set collections and used in gene-set analysis by piano, based on the gene q-values and fold-changes from the mice fed HFD-P vs. HFD-S comparison for each tissue.

#### Analysis of real-time Quantitative Polymerase Chain Reaction Assays in WAT

Gene expression of macrophage markers in WAT was analyzed by a one-way ANOVA followed by Tukey’s post hoc test and data were expressed as arithmetic means + standard errors of the means (SEM).

#### Analysis of WAT and LIV

SPL and LIV weight were analyzed using a two-way ANOVA, with experimental days as nuisance factors. The data was expressed as estimated marginal means + SEM. CLS/μm2 analyzed using the nonparametric Kruskal-Wallis test followed by Mann-Whitney tests. Because the values for some of the samples were set to zero, nonparametric tests were used; therefore, no measure of the spread of data for CLS is presented. These data are presented as scatter plots with a line indicating the median. ORO staining of the LIV was analyzed using two-way ANOVA followed by Tukey’s multiple comparisons test.

#### Analysis of metabolic parameters

B-glucose and OGTT area under the curve (AUC) were analyzed using a one-way ANOVA followed by Tukey’s post hoc test and data were expressed as means + SEM. The HOMA-IR index was logarithmic transformed to ensure normal distribution of data and analyzed using a one-way ANOVA followed by Tukey’s post hoc test with data was expressed as geometric means + geometric SEM.

## Results

### The diets affected the tissues differently

**Fig 1:**
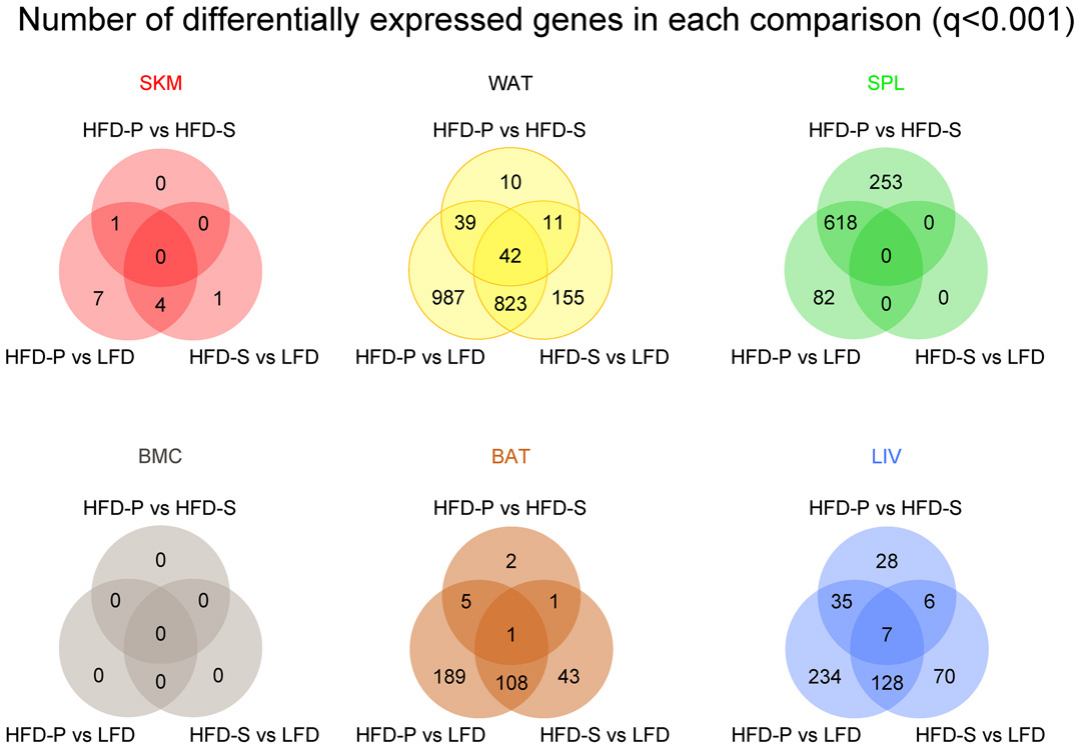
Venn diagrams showing the number of differentially expressed genes (*q*<0.001) in each diet-comparison for each tissue. SKM and BMC showed little transcriptional response to diet, whereas hundreds of genes were affected in the other four tissues. In BAT and WAT there was mainly an effect of HFD. In SPL there was mainly an effect of HFD-P. In LIV there was an influence of both HFDs *per se*, but also additionally, separate effects of HFD-P and HFD-S. SKM, skeletal muscle; BMC, bone marrow cells; BAT, brown adipose tissue; WAT, white adipose tissue; HFD, high fat diet; SPL, spleen; HFD-P high fat diet rich in polyunsaturated fatty acids; LIV, liver; HFD-S, high fat diet rich in saturated fatty acids.

To get an overall assessment of the effects of the diets on the different tissues, we investigated differentially expressed genes in three comparisons: mice fed HFD-S *vs*. LFD, HFD-P *vs*. LFD and HFD-P *vs*. HFD-S. Fig 1 displays Venn diagrams showing the number of differentially expressed genes for each tissue and comparison at a cutoff of *q*<0.001. The tissues were affected differently by the diets at the transcriptional level. SKM and BMC were relatively inert to the diets, whereas the two adipose tissues (WAT and BAT) were mainly affected by HFDs *per se* (both HFD-S and HFD-P). The most dramatic effect of HFD-P on tissue transcriptomics was observed in spleen, an immune tissue. Remarkably, the spleen was barely affected at all by HFD-S compared with LFD. In contrast, the LIV, primarily a metabolic tissue, exhibited a mixed response to HFD-S as well as HFD-P compared with LFD (Fig 1).

### HFDs caused WAT to adopt a pattern similar to immune system tissues and PUFA had specific effects in SPL

**Fig 2:**
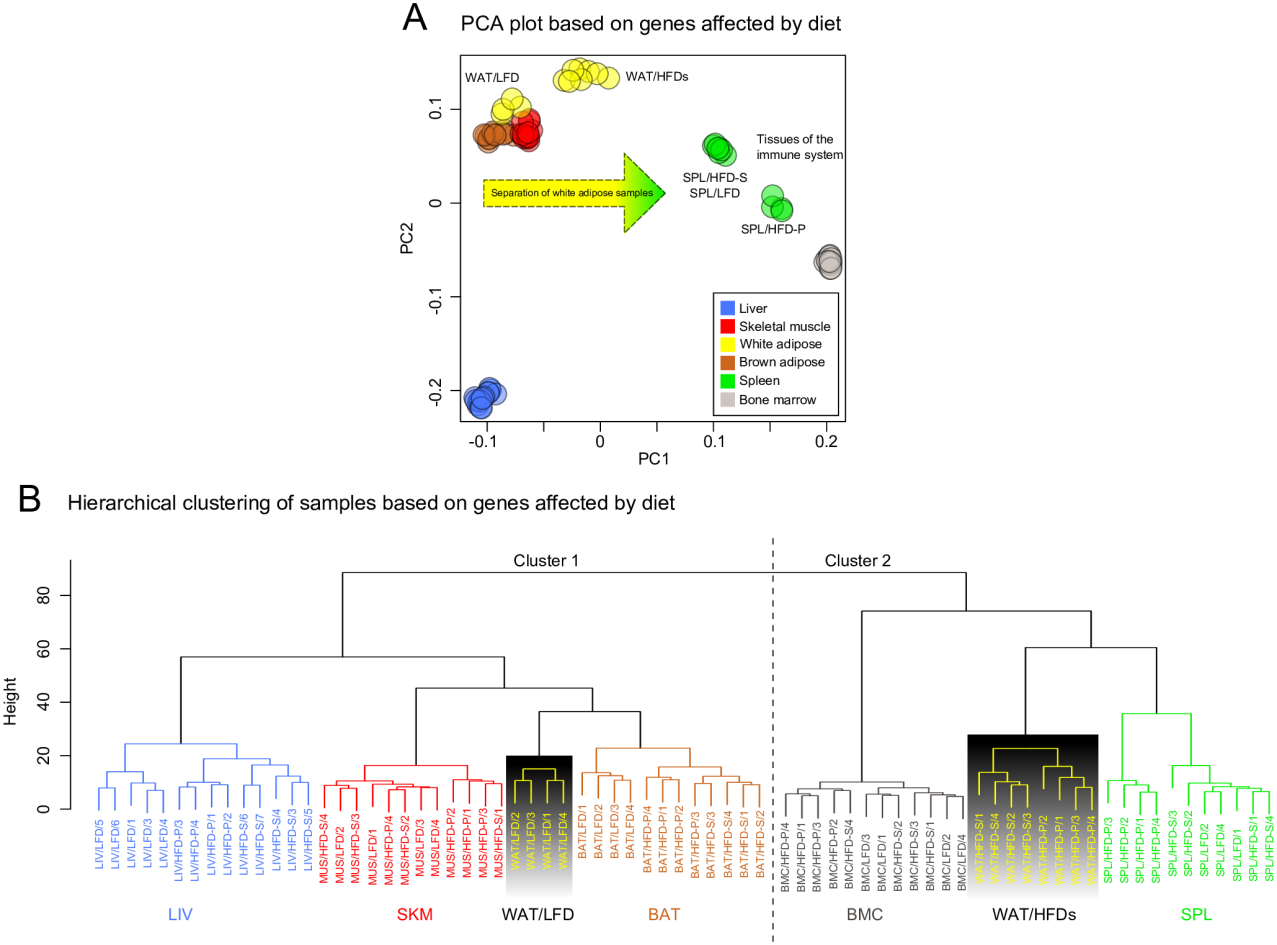
(A) PCA plot based on normalized gene expression data for differential expressed genes that were highly significant (*q*<1e-7) in any diet comparison and any tissue. As expected samples clustered tissue-wise, but interestingly, the WAT/HFD samples separated from the LFD samples and appeared closer to the two immune tissues SPL and BMC. It is also apparent in SPL, the HFD-P samples separated from the LFD and HFD-S. These observations suggest that diet effects on tissue transcriptomes were mainly observed in WAT and SPL, influenced by HFD *per se* in the former and by HFD-P specifically in the latter. (B) A hierarchical tree of the samples was constructed based on the same genes used in the PCA plot. Here, the separation of the WAT samples was clear as they divided into the two main clusters, the LFD samples together with LIV, SKM and BAT, and the HFD samples with BMC and SPL.

In order to investigate the general effect of diet across tissues, the differentially expressed genes that were highly significant (*q*<1e-7), in at least one of the tested comparisons, were used to cluster all samples in a PCA plot (Fig 2A). As expected, the PCA plot shows that samples from the same tissue clustered together. However, two interesting patterns appeared. First, HFD-P had a strong effect on gene expression in SPL. Secondly, both WAT/HFDs samples (*i*.*e*. WAT from mice fed HFD-S and HFD-P) were clearly separated from the WAT/LFD samples and seemed to be more closely related to the samples from the immune system tissues SPL and BMC than the WAT/LFD samples.

In order to further explore these interesting patterns, a hierarchical clustering of the samples was performed based on the same selection of data as described above for the PCA plot. The hierarchical tree shown in Fig 2B separated the samples in two main clusters: Cluster 1 consisting of the SKM, BAT and LIV samples, and cluster 2 consisting of the BMC and SPL samples. In agreement with the PCA plot, the WAT samples were separated between these two clusters: The WAT/LFD samples in cluster 1, together with the metabolic tissues, and the WAT/HFD-P and WAT/HFD-S samples, together with the immune-related tissues, in cluster 2. This suggests that HFDs, regardless of the fatty acid composition, causes transcriptional changes in WAT that are similar to the transcriptional patterns of the immune organs BMC and SPL. In addition to the PCA plot, a clear HFD-P effect on the SPL transcriptome was also observed by the hierarchical clustering, albeit not as dramatic as the separation of the WAT samples.

### Spleen transcriptome was affected almost exclusively by PUFA

**Fig 3:**
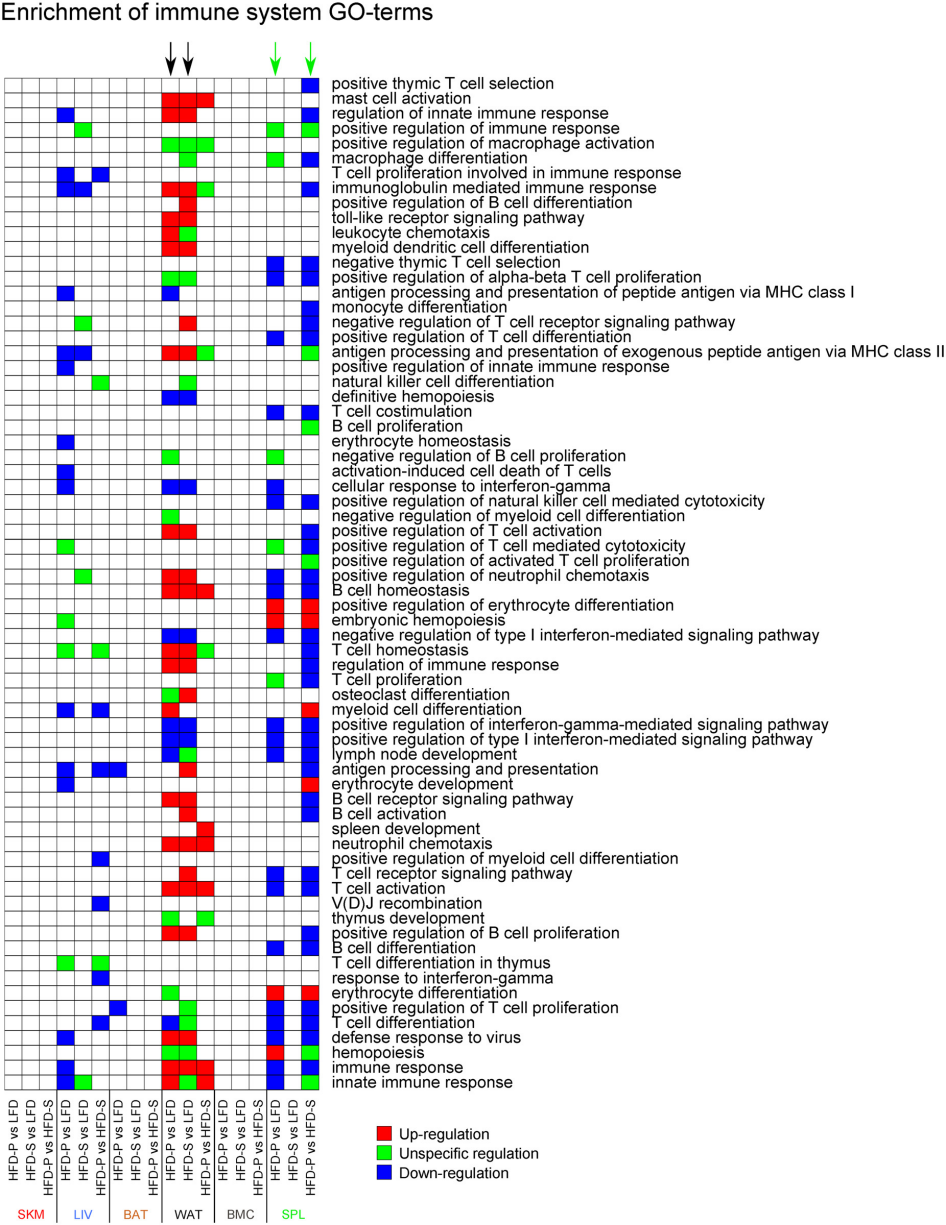
A heatmap showing significant (*q*<0.05) immune system GO-terms for the different diet comparisons across all tissues. Changes in diet affected immune system related gene expression in primarily WAT and SPL. HFDs resulted in major changes in WAT (indicated by black arrows) whereas HFD-P, specifically, resulted in the changes observed in SPL (indicated by green arrows). The figure is based on the adjusted non-directional p-values (S1–S6 Tables). Color indicates GO-terms affected by transcriptional up-regulation (red) or down-regulation (blue). Green indicates a significant yet unspecific regulation and white indicates no significant regulation. WAT, white adipose tissue; SPL, spleen; HFDs, high fat diets; HFD-P high fat diet rich in polyunsaturated fatty acids.

In order to investigate dietary effects on expression of genes associated with immune related functions, we carried out a gene-set analysis of immune system GO-terms [23]. Interestingly, the effect of dietary fatty acids in SPL differed from that of the other tissues. In SPL, HFD-S had a similar effect as LFD, whereas HFD-P induced a transcriptional pattern distinct from the other two diets. Thus, the specific fatty acid composition of HFD-P, rather than the increase in fat amount, seemed to be the main triggering factor for the transcriptional changes in SPL (Fig 3). The affected genes were mostly involved in the adaptive immune system, and to a lesser extent, in the innate immune system and erythrocyte control. HFD-P down-regulated the adaptive and innate immune system and up-regulated erythrocyte development.

### Major regulation of immune-related genes in WAT/HFDs

The gene-set analysis of immune system GO-terms revealed that in WAT, differentially expressed genes were involved in both the innate and the adaptive immune system (Fig 3). As seen in Fig 3 the major effects on immune related GO terms in WAT are by both the HFDs, regardless of fatty acid composition. In general, HFDs up-regulated both the innate and the adaptive immune system (HFD-P *vs*. LFD and in HFD-S *vs*. LFD). However, HFDs also down-regulated specific GO-terms related to interferon mediated signaling, including both type I and type II interferon (HFD-P *vs*. LFD and HFD-S *vs*. LFD). In addition, HFD-P also down-regulated T cell differentiation compared with mice fed LFD (HFD-P *vs*. LFD). Only a few GO-terms were affected when comparing HFD-P *vs*. HFD-S, these also included an up-regulation of both innate and adaptive immune responses (Fig 3).

**Fig 4:**
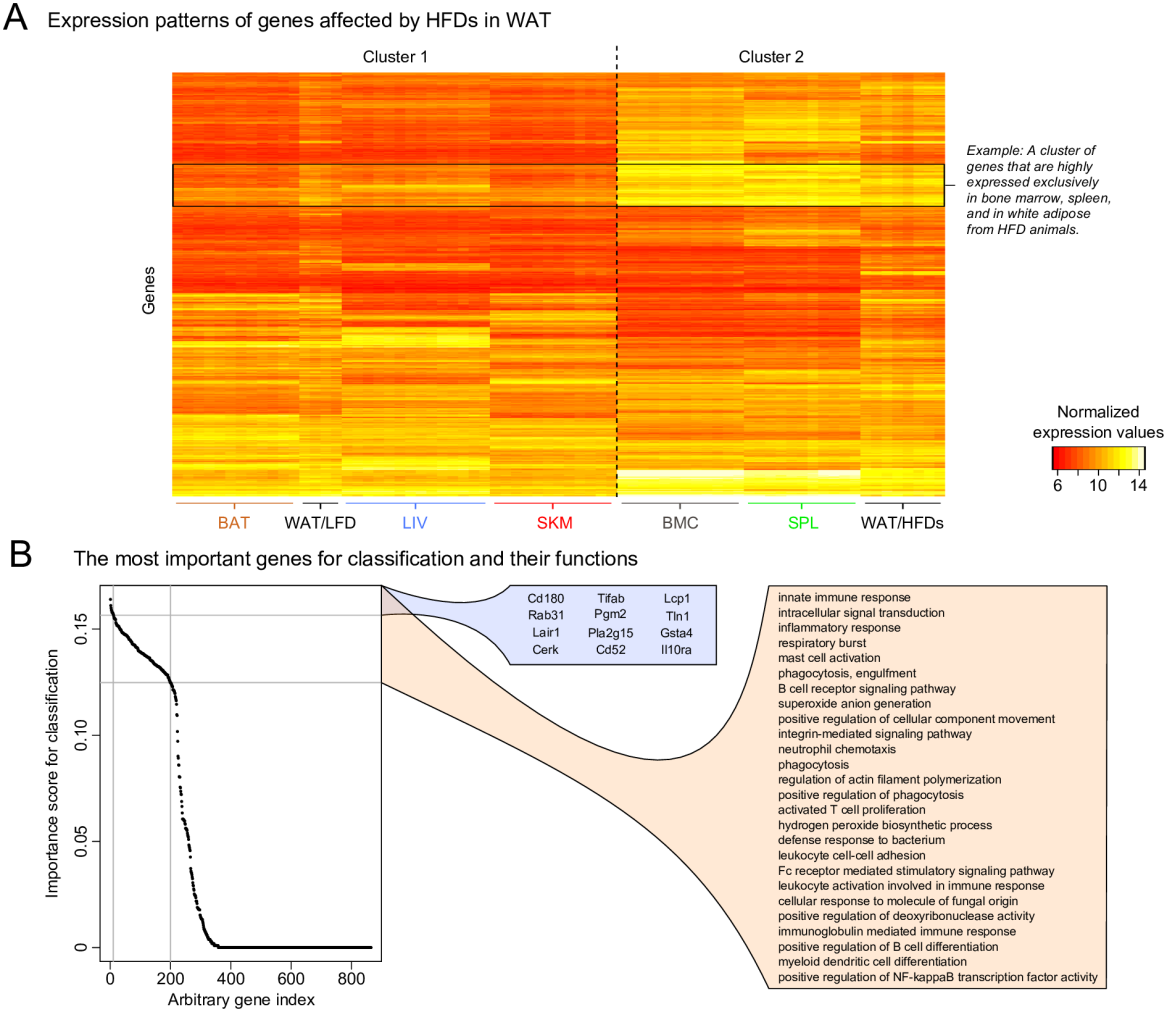
(A) A heatmap of expression values from all samples for the subset of genes that were significantly changed (*q*<0.001) in WAT by HFD (HFD-S *vs*. LFD or HFD-P *vs*. LFD). Again, the samples divided into the same two main clusters as in Fig 2B (hierarchical tree not shown). The heatmap enables the identification of groups of genes that show similar expression values in the WAT/HFD samples as in the BMC and SPL samples and differ from the expression values in WAT/LFD and the remaining tissues. (B) In order to extract the genes in an unbiasedly manner that drive the separation of the WAT samples into the two clusters, we performed a sample classification using supervised learning, based on the same genes as in (A). In this process each gene is scored based on its influence on classification. The plot shows the ordered scores and it can be seen that roughly 200 genes are important for the separation into the two clusters, whereas the remaining genes have little or no importance. The top box shows the 12 most important genes and the bottom box shows the enrichment of GO-terms (Biological Processes) for the top 200 genes, mainly illustrating their involvement in immune system related processes. PCA, principle component analysis; WAT, white adipose tissue; HFD, high fat diet; LFD, low fat diet; SPL, spleen; BMC, bone marrow cells; HFD-P high fat diet rich in polyunsaturated fatty acids; HFD-S, high fat diet rich in saturated fatty acids; LIV, liver; SKM, skeletal muscle; BAT, brown adipose tissue; GO, gene ontology.

In light of the observed clustering of WAT/HFDs samples with SPL and BMC we wanted to investigate what genes were driving these patterns. To do this we constructed a heatmap (Fig 4A) of gene expression signals from all samples, specifically for the subset of genes that were significantly (*q*<0.001) differentially expressed in WAT between either mice fed HFD-P *vs*. LFD or HFD-S *vs*. LFD. The heatmap can help to identify if there is a characteristic gene expression pattern across tissues that distinguish the two main clusters. For example, a subset of genes that shows a particularly high expression levels in the samples of cluster 2 when compared with cluster 1. It is clear from the heatmap that there is a specific set of genes that were responsible for the similarity between the WAT/HFDs, SPL and BMC samples.

To further characterize the HFD-regulated genes in WAT causing the separation between the two clusters, we performed sample classification using supervised learning (random forests model). Briefly, the expression signals of the genes in the heatmap (*i*.*e*. HFDs-regulated genes in WAT) were used as input to the classification model so that each sample was predicted to belong to either cluster 1 or 2. This enabled us to identify the genes that were most important for classification of the two clusters thus driving the separation of the WAT samples. The importance scores are depicted in Fig 4B (sorted by decreasing importance) showing that roughly 200 genes had high impact on the classification into the two clusters, whereas the remaining roughly 600 genes had little importance. In order to identify the general biological functions these 200 genes represent, we performed a GO-term (all Biological Processes) overrepresentation analysis, using the hypergeometric test. The most significantly enriched (*q*<0.05) functions are presented in the lower box. These were to a large extent represented by immune-related processes and out of these 200 genes, 54 belonged to the cluster of genes marked in the heatmap in Fig 4A. The upper box contains the top 12 genes (in terms of classification importance) and can be seen as drivers of the separation of the WAT samples. Since the individual importance scores of the top 200 genes are quite similar, the list of the top 12 genes was estimated by repeating the classification described above 100 times.

### Increased expression of macrophage markers and CLS in WAT/HFD-S

**Fig 5:**
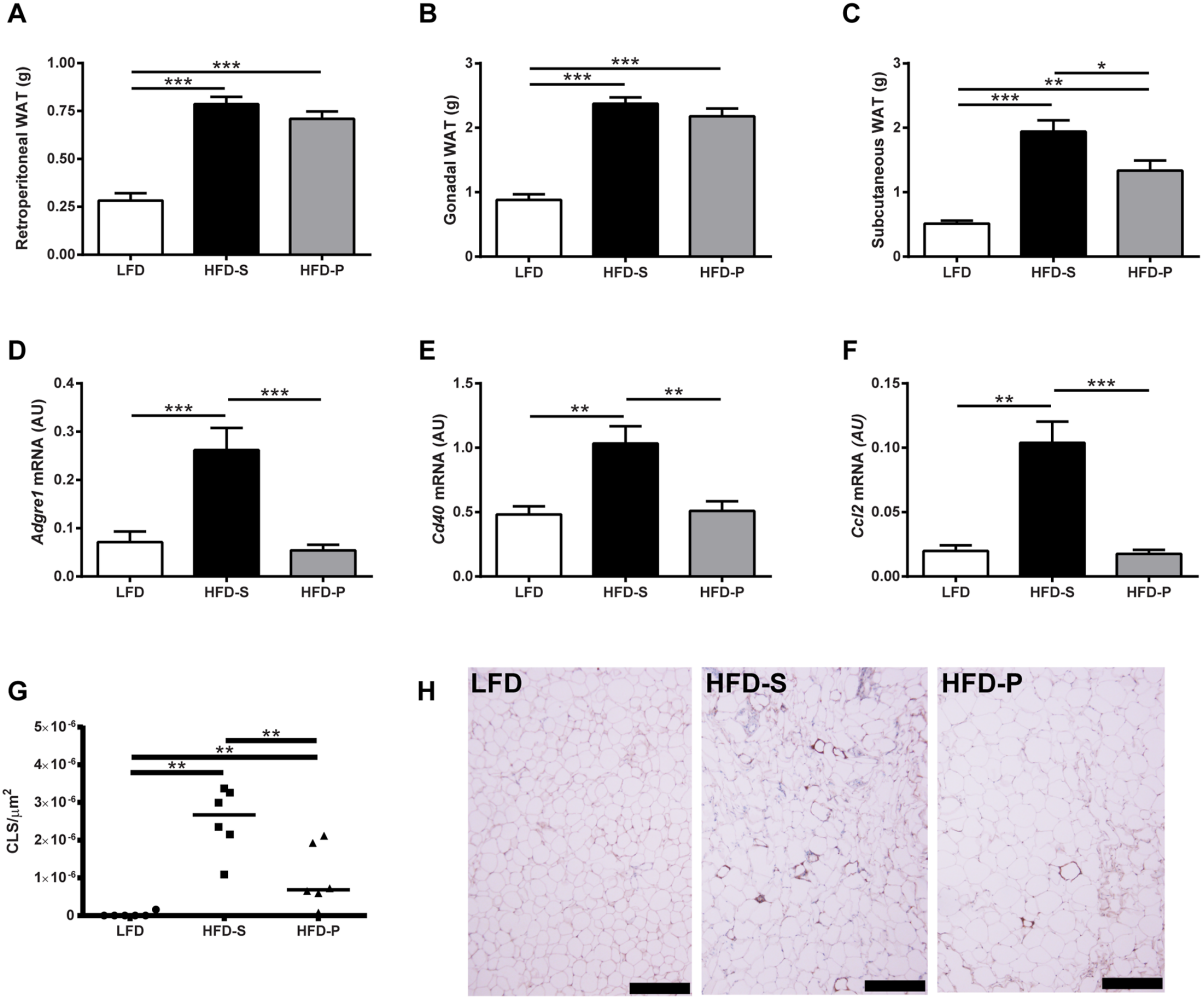
(A) Organ weight of retroperitoneal WAT and (B) gonadal and (C) subcutaneous WAT from mice fed LFD, HFD-S and HFD-P. One-way ANOVA with Tukey’s post-hoc test, *n* = 8 mice per group. Data are shown as mean + SEM. mRNA levels of macrophage marker (D) *Adgre1* (F4/80), M1 markers (E) *Cd40* (CD40) and (F) *Ccl2* (MCP-1) in retroperitoneal WAT from mice fed LFD HFD-S and HFD-P. One-way ANOVA with Tukey’s post-hoc test, *n* = 8–10 mice per group. Data are shown as mean + SEM. (G) Number of CLS/μm^2^ in WAT from mice fed LFD, HFD-S and HFD-P. CLS was analyzed by Kruskal-Wallis test (global *p* = 0.001) followed by Mann-Whitney test, with *n* = 6 mice per group. Data are shown as scatter plots with the line indicating the median. (H) Representative micrographs showing F4/80 staining in WAT from mice fed LFD, HFD-S and HFD-P. Scale bar represents 200 μm. *, **, ***: *p*<0.05, <0.01 and <0.001 respectively. WAT, white adipose tissue; LFD, low fat diet; HFD-S high fat diet rich in saturated fatty acids; HFD-P high fat diet rich in polyunsaturated fatty acids; CLS, crown like structures.

To be able to compare our study with previous studies investigating effects of fatty acid composition we investigated adipose tissue depots and their immunological phenotype. Both mice fed HFD-S and HFD-P had increased visceral (retroperitoneal and gonadal) and subcutaneous (inguinal) WAT weights compared with mice fed LFD. For retroperitoneal and gonadal WAT, there was no difference between mice fed HFD-S and HFD-P (Fig 5A and 5B). However, for the subcutaneous WAT, mice fed HFD-S had increased weight compared with HFD-P (Fig 5C).

To further investigate the immunological phenotype displayed by WAT after HFD, macrophage markers were analyzed with quantitative PCR. Mice fed HFD-S had higher expression of the macrophage marker *Adgre1* (F4/80) in WAT, as well as M1 markers *Cd40* (CD40) and *Ccl2* (MCP-1), compared with both mice fed HFD-P and LFD (Fig 5D, 5E and 5F). There was no difference in the expression of *Adgre1*, *Cd40* or *Ccl2* between mice fed LFD and HFD-P (Fig 5D, 5E and 5F). We further characterized the infiltration of macrophages into WAT by quantifying the numbers of CLS. Mice fed HFD-S had increased number of CLS/μm^2^ in WAT compared with mice fed LFD and HFD-P (Fig 5G). Mice fed HFD-P had increased number of CLS/μm^2^ in WAT compared with mice fed LFD. Representative micrographs of WAT are presented in Fig 5H.

### HFD-P corrects the metabolic phenotype induced by HFD-S

**Fig 6:**
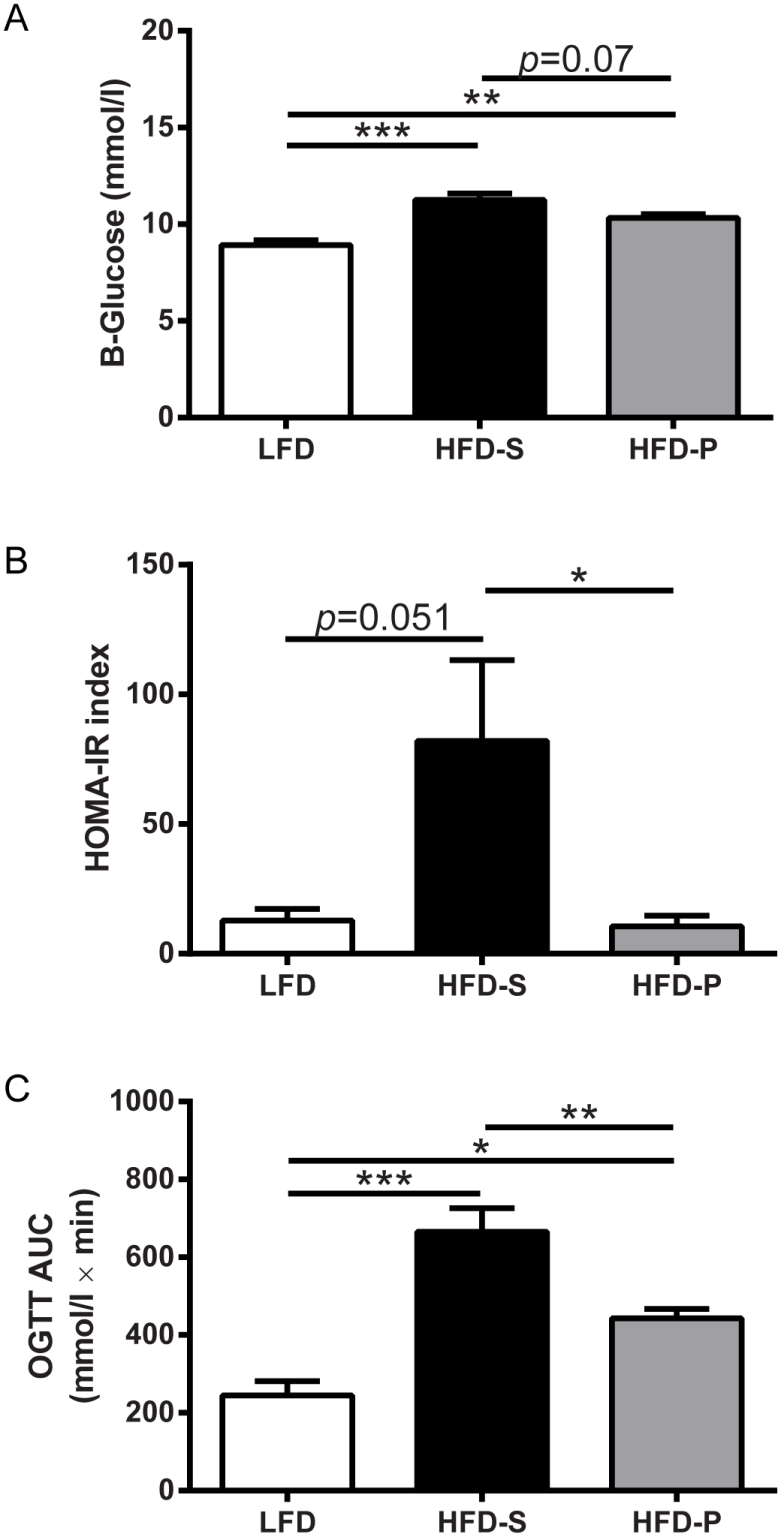
(A) Fasting B-Glucose levels, (B) HOMA-IR index and (C) OGTT AUC from mice fed LFD, HFD-S and HFD-P. For (A) and (C) data are shown as mean + SEM, for (B) data are shown as back transformed geometric mean + geometric SEM. One-way ANOVA with Tukey post-hoc test, *n* = 5–6 mice per group. HOMAR-IR, homeostasis model assessment of insulin resistance; OGTT, oral glucose tolerance test; AUC, area under the curve; LFD, low fat diet; HFD-S, high fat diet rich in saturated fatty acids; HFD-P, high fat diet rich in polyunsaturated fatty acids (HFD-P).

To investigate the metabolic phenotype induced by the different diets, fasting blood glucose concentration, HOMA-IR index and OGTT were measured. Fasting blood glucose levels were higher in mice fed HFD-S and HFD-P compared with mice fed LFD (Fig 6A). There was no difference between mice fed HFD-S and HFD-P. Further, HOMA-IR index was higher in mice fed HFD-S compared with mice fed LFD and HFD-P, whereas there was no difference between mice fed HFD-P and LFD (Fig 6B). The glucose levels at different time points (0–120 min) during the OGTT were measured and the AUC was calculated. Mice fed HFD-S had higher OGTT AUC compared with mice fed LFD and HFD-P (Fig 6C). Mice fed HFD-P had higher OGTT AUC compared with mice fed LFD.

### LIV transcriptomic changes showed a mixture of HFDs and HFD-P effects

As shown by the Venn diagram (Fig 1) LIV were affected by both HFDs. Both HFD-P and HFD-S regulated immune related GO-terms although not as dramatic as seen in SPL and WAT (Fig 3). In fact, the immune system GO-terms that were specifically affected by HFD-P were higher in number and included down-regulation of both the innate and the adaptive immunity (HFD-P *vs*. LFD and HFD-P *vs*. HFD-S, Fig 3).

**Fig 7:**
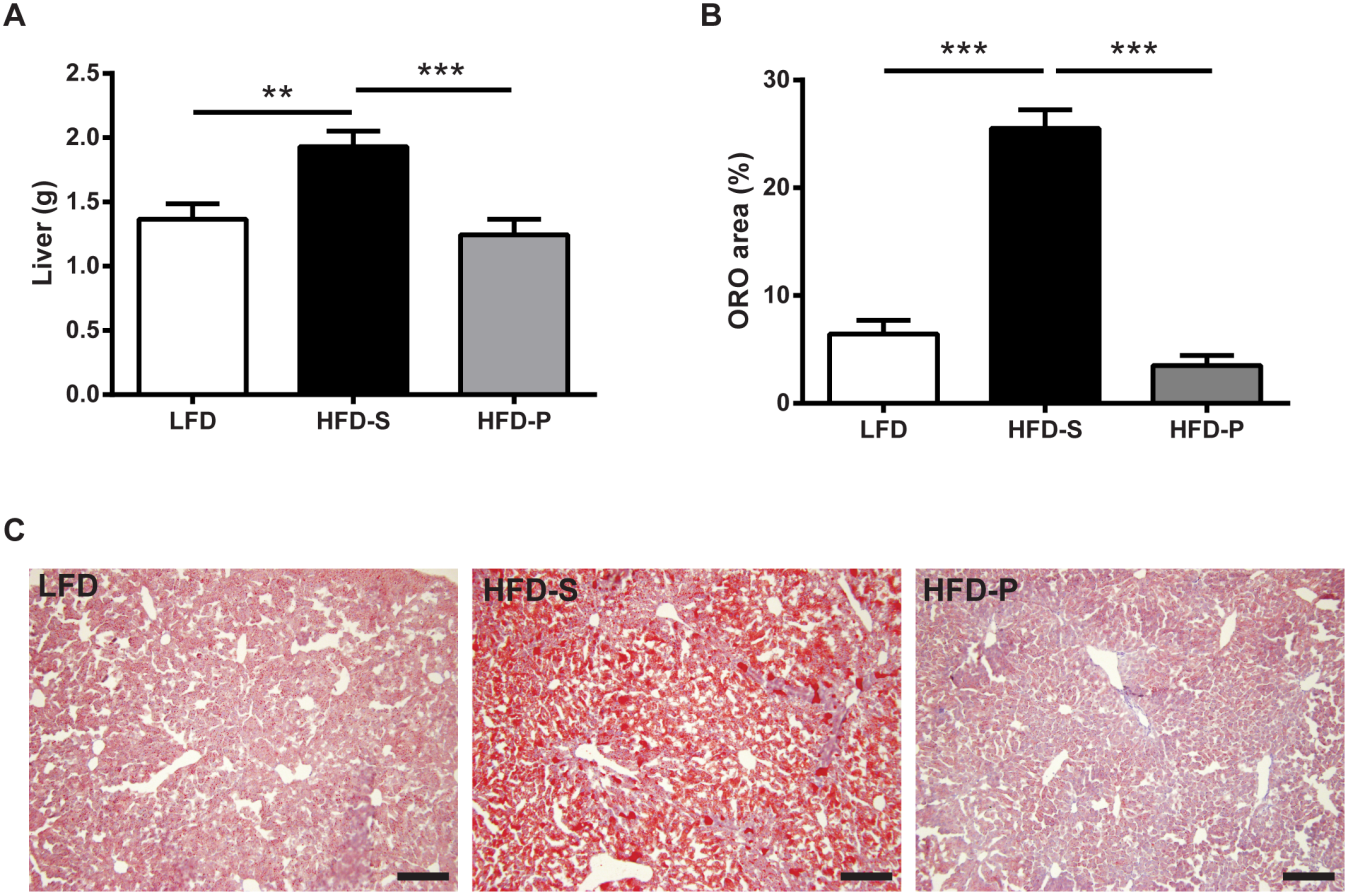
(A) Organ wight of LIV, (B) percentage ORO positive areas in LIV and (C) representative micrographs from mice fed LFD, HFD-S or HFD-P. Scale bar represents 200 μm. For (A) two-way ANOVA with experiment as nuisance factor, *n* = 10 mice per group. Data are shown as estimated marginal mean + SEM. For (B) two-way ANOVA followed by Tukey’s multiple comparisons test. **, ***: *p*<0.01, <0.001 respectively. LIV, liver; ORO, oil red O; LFD, low fat diet; HFD-S, high fat diet rich in saturated fatty acids; HFD-P, high fat diet rich in polyunsaturated fatty acids (HFD-P).

Further, mice fed HFD-S had increased LIV weight compared with mice fed HFD-P and LFD. There was no difference in liver weight between mice fed LFD and HFD-P (Fig 7A). ORO staining of the LIV revealed that mice fed HFD-S had distinctly more fat inclusions in their hepatocytes compared with LIV from mice fed HFD-P and LFD (Fig 7B and 7C).

### BAT, SKM and BMC transcriptomics were relatively inert to the different diets

Although BAT was mainly affected by HFDs, the immune system GO-terms were not highly regulated in BAT. In fact, only 2 immune system GO-terms were significantly down-regulated by HFD-P (HFD-P *vs*. LFD), *i*.*e*. “antigen processing and presentation” and “positive regulation of T cell proliferation” (Fig 3). In both SKM and BMC, none of the immune system GO-terms were regulated (Fig 3).

### NFκB was affected by HFDs

In order to investigate whether the expression of genes under the control of the transcription factor NFκB was affected by the different diets, we performed a gene-set analysis. This revealed that in WAT, SPL and LIV NFκB was affected by the different diets, *i*.*e*. NFκB target genes displayed a general down-regulation in mice fed HFD-P *vs*. HFD-S, especially in the SPL (S2 Fig).

## Discussion

In this study, we investigated the transcriptome profiles of six different tissues after an 8 week feeding period, where the mice had been fed LFD, HFD-S or HFD-P. We demonstrated that dietary PUFA suppressed expression of immune related genes in spleen, whereas there were negligible effects of HFD-S compared with LFD. In addition, regardless of fat composition, HFD *per se* induced a specific gene expression profile in WAT compared with LFD. Further, HFD-P corrected the metabolic phenotype induced by HFD-S. These results are of interest in relation to our previous findings from this experimental model where the total amount and the dietary fatty acid composition affected immune function as well survival in septic infections [7,8,24].

### Dietary fat composition affected the transcriptome of SPL but not BMC

The present results clearly show that dietary SFA and PUFA differently affected the transcriptome of SPL, a well-recognized immune organ. Surprisingly, this effect was more dramatic than the SFA and PUFA effects on the LIV, SKM, WAT and BAT transcriptomes, all recognized as primarily metabolic organs. This finding is well in line with our previous reports showing that the dietary fatty acid composition can greatly influence the immune system, including its capacity to clear certain bacteria [7]. Moreover, the immune capacity to clear bacteria may be lower in mice fed HFD-S than in those fed LFD [24].

The mechanism by which PUFA, and especially ω-3 PUFA can affect the immune system is not clear. One possibility is that ω-3 PUFA acts directly, by decreasing the content of arachidonic acid, the precursor of pro-inflammatory eicosanoids, or by being the precursor of resolvins, some anti-inflammatory fatty acid derivatives [3]. ω-3 PUFA may also act by altering transcription factor activation [3]. Further, PUFA in the cell membrane may increase plasma membrane fluidity [25], and thereby improve the phagocytosis capacity [26].

The two immunological tissues included in the current analysis (BMC and SPL) displayed clearly differentiated response to the diets. While gene expression in BMC was barely affected at all, there was a significant effect on SPL in mice fed HFD-P, but not in those fed HFD-S. The lack of dietary effects in the BMC was surprising, but it should be noted that the cut-off for significant difference was set rather high (*q*<0.001). We have previously reported that mice fed HFD-P have a higher frequency of neutrophils in the bone marrow at an uninfected state and show a better immune response during an *S*. *aureus*-induced sepsis compared with mice fed HFD-S [7].

In SPL, HFD-P affected gene expression related to both the innate and the adaptive immune system. Interestingly, according to the GO classification, both arms of the immune system, innate and adaptive immune system, were down-regulated in SPL from mice fed HFD-P compared with mice fed HFD-S and LFD. This is in line with the previously reported anti-inflammatory responses mediated by PUFA in peripheral blood mononuclear cells [27].

Genes associated to erythrocyte control were up-regulated in SPL from mice fed HFD-P compared with mice fed HFD-S and LFD. However, the erythrocyte concentration in the blood did not differ between the groups (data not shown). This differentially expression of genes regulating erythrocyte development and differentiation may reflect an increased turnover without affecting their concentration in blood [28].

### HFDs induced an immunological phenotype in WAT

The primary purpose of white adipose tissue is energy storage; however, in the hierarchical clustering analysis, the WAT from mice fed HFDs clustered with the immunological tissues BMC and SPL. Hence, in mice fed HFDs, WAT developed an immunological phenotype. This is in line with previous work showing that hypertrophied WAT secretes pro-inflammatory agents promoting systematic inflammation, and that there is macrophage recruitment to WAT in obese individuals [29,30]. Indeed, in the current study WAT from mice fed HFD-S had higher gene expression of macrophage marker *Adgre1* (F4/80), as well as the M1 markers *Cd40* (CD40) and *Ccl2* (MCP-1), compared with mice fed LFD. This was further supported by the increase in CLS in WAT from mice fed HFD-S. Interestingly, despite the similar weight of the visceral WAT in mice fed HFD-S as in mice fed HFD-P, mice fed HFD-P did not have increased macrophage markers or CLS. Additionally, mice fed HFD-P had lower HOMA-IR index and OGTT AUC compared with mice fed HFD-S. These findings confirm previous data that adipose tissue inflammation is prevented by dietary PUFA [12], as found in HFD-P, and that dietary PUFA can improve glucose homeostasis [31].

The general consensus has been that inflammation in white adipose tissue is deleterious and contributes to the metabolic syndrome. However, it was recently suggested that in order to maintain a healthy adipose tissue expansion and remodeling, a local acute adipocyte inflammation is crucial [32]. Adipose tissue expansion and remodeling is critical for protecting against deposition of lipids in other tissues, such as the liver. Indeed, in the current study we detected liver lipid deposition in mice fed HFD-S, but not in mice fed HFD-P. Hence, this indicates that PUFA maintain the expansion and remodeling of WAT [33].

### Immunological effects in LIV by HFDs

Besides the major role in metabolic regulation, the liver also has an important immunological role [34]. Indeed, LIV was affected by the two different HFDs in specific ways. Both HFDs led to a down-regulation of the immune system GO-terms, but the number of suppressed GO-terms was higher for mice fed HFD-P compared with mice fed HFD-S. Further, HFD-P down-regulated several GO-terms involved in immune responses, suggesting an anti-inflammatory effect of HFD-P in the liver. ORO staining showed that the LIV from all groups (LFD, HFD-P, and HFD-S) had lipid droplets in the hepatocytes; however, the lipid droplets were clearly larger and more numerous in mice fed HFD-S, confirming previous publications [11,35]. The present results are also in line with earlier studies showing that HFD-P favorably can affect lipid metabolism in the liver and decrease lipid deposits in this organ [36,37].

### SKM was inert to the different diets

Skeletal muscle is the largest insulin-sensitive tissue in the body. When this tissue becomes insulin resistant this will contribute to the development of the metabolic syndrome [38]. There are several suggested mechanisms behind the reduced insulin signaling and intramuscular lipid accumulation is one of them. HFD-S may increase intramuscular lipid accumulation, whereas HFD-P can reverse this effect by influencing the expression of genes involved in lipid metabolism [39]. In the current study, SKM (gastrocnemius) was surprisingly inert to the dietary changes in terms of immune related GO-terms, *i*.*e*. none of the immune related GO-terms were affected by the dietary changes.

It has previously been reported that mice fed a diet rich in PUFA (docosahexaenoic acid) has an up-regulation of genes associated with glucose use and down-regulation of genes associated with systemic inflammatory status [40]. We did not see these genes affected in the current study, possibly because of analysis of different muscle types [39].

### Dietary fatty acids affect the transcription factor NFκB

Both SFA and PUFA have indirect effects on the immune system by influencing the activity of transcription factors. For example, SFA and ω-3 PUFA affect NFκB in opposite ways [1,3,41]. NFκB is a major transcription factor regulating the transcription of pro-inflammatory cytokines, like interleukin 1-beta, interleukin-6, interferon-γ, monocyte chemotactic protein-1, and tumor necrosis factor [3,42]. ω-3 PUFA decrease endotoxin-induces activation of NFκB in monocytes and macrophages whereas SFA has the opposite effect and enhances NFκB activation in macrophages in white adipose tissue [1] and immune cells [41]. Indeed, in the current study NFκB was influenced by HFD-P compared with HFD-S in WAT, SPL and LIV, as shown by the general down-regulation of NFκB target genes induced by HFD-P. This could also explain the higher expression of M1 macrophage markers *Cd40 and Ccl2* in WAT from mice fed HFD-S compared with both mice fed HFD-P and LFD. Another transcript factor that has been reported to be affected in opposite directions by SFA and PUFA is peroxisome proliferator-activated receptor γ [3,43,44]. However, this factor was associated with transcriptional down-regulation of its target genes, by both the HFDs in our experiment (data not shown).

### Total amount of fatty acids *vs*. the composition of the fatty acids

The amount of dietary fatty acids affects gene expression, as confirmed in the current study where mice fed two different HFDs showed a distinct gene expression pattern in the six tissues investigated compared with mice fed LFD. However, the fatty acids composition *per se* can greatly influence the transcriptome profile [3,41]. Moreover, the total amount of fatty acids are less important for the development of cardiovascular disease and type 2 diabetes mellitus compared with the composition of the fatty acids [5,6]. This is in line with the current study where the two isocaloric HFDs, HFD-S and HFD-P, induced two distinct gene expression patterns in tissues of importance for regulation of metabolic and immune functions. These findings on the gene expression patterns demonstrate the importance of investigation of composition as well as total amount of dietary fatty acids.

## Conclusions

In conclusion, our study shows that dietary fatty acid content and composition have specific effects in different tissues. To the best of our knowledge, we show for the first time that dietary PUFA markedly decreased the expression of immune stimulating genes in the spleen, while dietary SFA has no such effect.

## Supporting Information

S1 Fig PCA-plots for the individual tissues. PCA-plots for the different tissues showing one SPL/LFD sample as an outlier. PCA, principle component analysis; LFD, low fat diet; HFD-S, high fat diet rich in saturated fatty acids; HFD-P, high fat diet rich in polyunsaturated fatty acids; SKM, skeletal muscle; BMC, bone marrow cells; WAT, white adipose tissue; BAT, brown adipose tissue; SPL, spleen; LIV, liver.
(TIF)

S2 Fig Heatmap for enrichment of NfκB gene-sets in the 6-different tissues. Heatmaps showing the enrichment of differentially expressed genes (in mice fed HFD-P *vs*. HFD-S) for gene-sets of NFKB target genes. NFκB, nuclear factor kappa-light-chain-enhancer of activated B cells; HFD-S, high fat diet rich in saturated fatty acids; HFD-P, high fat diet rich in polyunsaturated fatty acids; SKM, skeletal muscle; BMC, bone marrow cells; WAT, white adipose tissue; BAT, brown adipose tissue; SPL, spleen; LIV, liver.
(TIF)

S1 Table Comparison of Immune related GO-terms for all diets in SPL. (XLSX)

S2 Table Comparison of Immune related GO-terms for all diets in WAT. (XLSX)

S3 Table Comparison of Immune related GO-terms for all diets in LIV. (XLSX)

S4 Table Comparison of Immune related GO-terms for all diets in BAT. (XLSX)

S5 Table Comparison of Immune related GO-terms for all diets in SKM. (XLSX)

S6 Table Comparison of Immune related GO-terms for all diets in BMC. (XLSX)
