# Supplementary figures and images for: Six Tissue Transcriptomics Reveals Specific Immune Suppression in Spleen by Dietary Polyunsaturated Fatty Acids
Source: PLoS One. 2016 May 11;11(5):e0155099. doi: 10.1371/journal.pone.0155099 (PMC4864434; doi:10.1371/journal.pone.0155099)

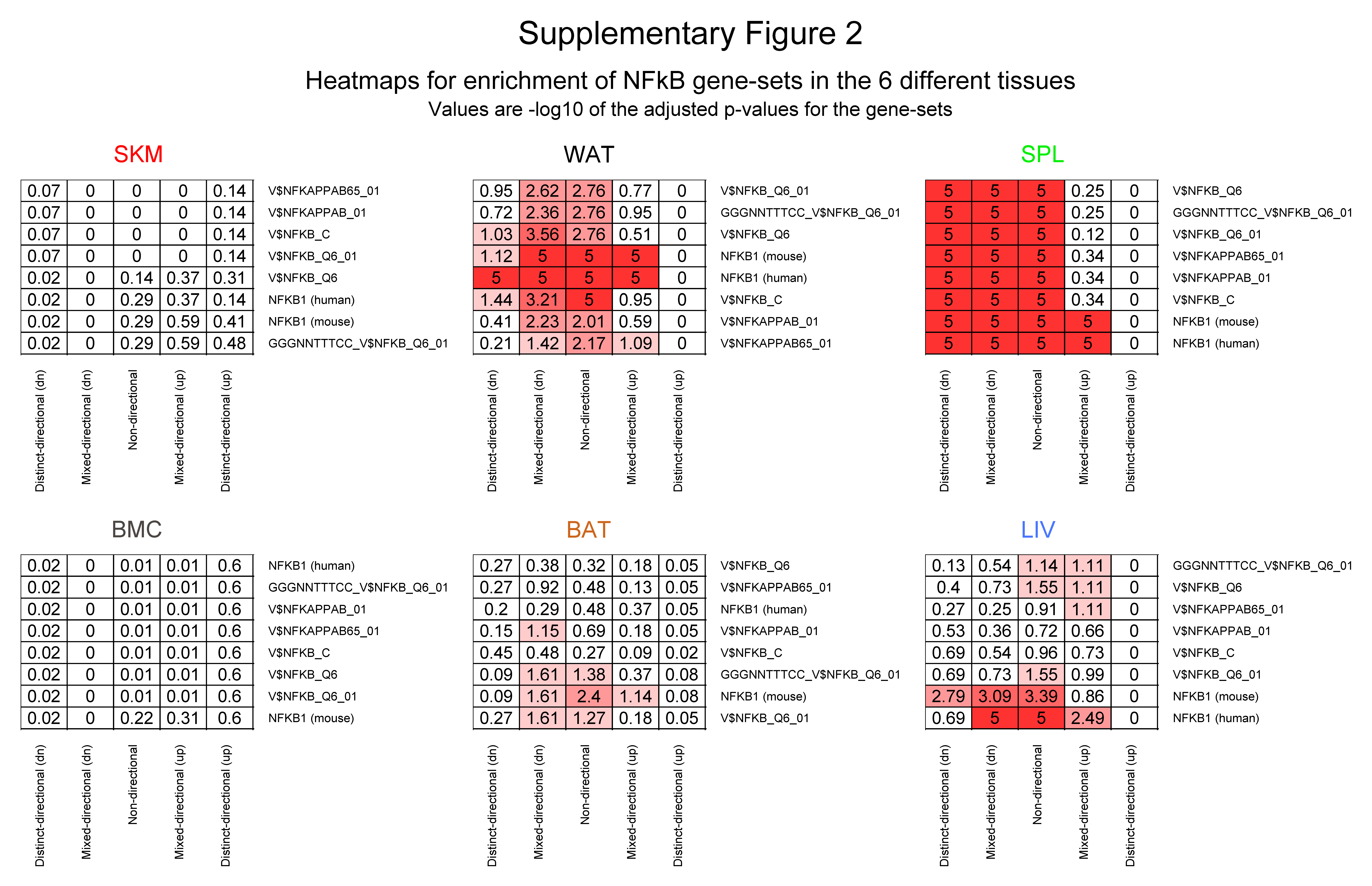

Supplement: S2 Fig — Heatmaps showing the enrichment of differentially expressed genes (in mice fed HFD-P vs. HFD-S) for gene-sets of NFKB target genes. NFκB, nuclear factor kappa-light-chain-enhancer of activated B cells; HFD-S, high fat diet rich in saturated fatty acids; HFD-P, high fat diet rich in polyunsaturated fatty acids; SKM, skeletal muscle; BMC, bone marrow cells; WAT, white adipose tissue; BAT, brown adipose tissue; SPL, spleen; LIV, liver. (TIF) [file pone.0155099.s002.tif]
